# Outstanding Reviewers for *Chemical Science* in 2016

**DOI:** 10.1039/c7sc90023f

**Published:** 2017-04-27

## Abstract

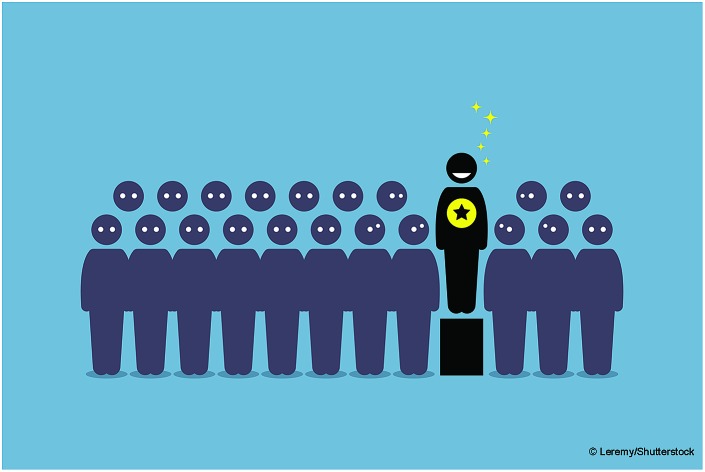
We would like to take this opportunity to highlight the Outstanding Reviewers for *Chemical Science* in 2016, as selected by the editorial team for their significant contribution to the journal.

## 

We would like to take this opportunity to thank all of *Chemical Science*'s reviewers, and in particular highlight the Outstanding Reviewers for the journal in 2016, as selected by the editorial team for their significant contribution to *Chemical Science*. Starting this year, we will be announcing our Outstanding Reviewers annually and each will receive a certificate to give recognition for their contribution. This new initiative will be featured across many of the journals published by the Royal Society of Chemistry. The reviewers have been chosen based on the number, timeliness and quality of the reports completed over the last 12 months.

 

## 

Professor Atsushi Fukuoka

 

## 

Hokkaido University

 

## 

Dr Gilles Gasser

 

## 

Chimie ParisTech, PSL Research University

 

## 

Professor Dirk Guldi

 

## 

Friedrich Alexander Universität

 

## 

Dr Christian Hackenberger

 

## 

Leibniz-Institut für Molekulare Pharmakologie im Forschungsverbund Berlin e.V. (FMP)

 

## 

Dr Takashi Hisatomi

 

## 

The University of Tokyo

 

## 

Dr Paul Knochel

 

## 

Ludwig-Maximilians-Universität

 

## 

Professor Jun Kubota

 

## 

Fukuoka University

 

## 

Professor Stefan Matile

 

## 

Universite de Geneve

 

## 

Professor Frank Wuerthner

 

## 

Universitaet Wuerzburg

 

## 

Professor Juyoung Yoon

 

## 

Ewha Womans University

 

## 

We would also like to thank the Chemical Science board and the General Chemistry community for their continued support of the journal, as authors, reviewers and readers.

 

## 

Philippa Hughes, Executive Editor

## 

Susan Weatherby, Editorial Production Manager

